# Technical note on an ovine model to study biomedical implants intended for maxillofacial reconstruction

**DOI:** 10.1111/vsu.70081

**Published:** 2026-02-22

**Authors:** Reza Sanaei, Ayda Farhoudi, Babatunde A. Ayodele, George Dimitroulis, Christina M. Murray, Helen M. Davies, Alastair J. Sloan, Charles N. Pagel

**Affiliations:** ^1^ Melbourne Veterinary School, Faculty of Science The University of Melbourne Parkville Victoria Australia; ^2^ MAXONIQ Melbourne Victoria Australia; ^3^ Melbourne Dental School, Faculty of Medicine, Dentistry & Health Sciences The University of Melbourne Parkville Victoria Australia

## Abstract

**Objective:**

To describe a repeated‐measures model permitting evaluation of up to four implants intended for maxillofacial applications in sheep.

**Animals:**

Two‐year‐old Merino wethers (*n* = 5).

**Methods:**

A retromandibular subparotid approach was developed through anatomical study of  atlases, predissected models and cadaveric experiments. A purpose‐designed surgical guide and test miniplates were produced to improve standardization among test implants. Five sheep were monitored for 6 months postoperatively to demonstrate the safety and practicality of the model.

**Results:**

All animals resumed food intake within 5 min of regaining the righting reflex. The only complication consisted of a mild unilateral swelling over the surgical site in two sheep, which resolved spontaneously within 8 weeks.

**Conclusion:**

The approach provided a safe and reliable surgical corridor to the mandibular ramus in sheep. Surgical site swelling should be assessed by ultrasonography to exclude implant involvement. This model may improve standardization and reduce animal numbers in studies evaluating maxillofacial implants.

## 1 INTRODUCTION

Maxillofacial tissues respond to implants in a different way from other skeletal sites. These bones demonstrate greater remodeling, faster healing, and lower mineralization rates.^1^, ^2^ Bone marrow stromal cells in this region show enhanced proliferative and osteogenic potential with delayed senescence.^3^ Maxillofacial soft tissues possess increased angiogenic and healing capacity but pose challenges such as greater bleeding risk and constant movement during mastication.^4^ A standardized animal model is therefore essential for evaluating biomaterials and devices intended for maxillofacial reconstruction.

**FIGURE 1:**
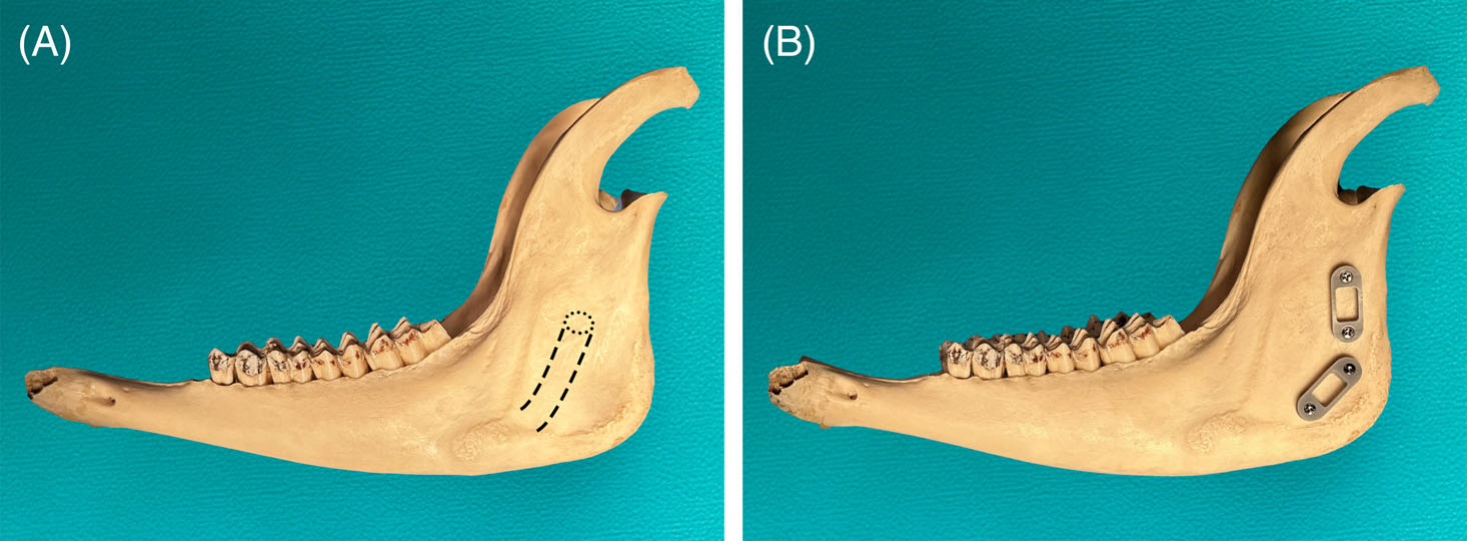
The lateral aspect of an ovine mandible. (A) The caudal and caudoventral regions of the masseteric fossa are consistently reasonably flat, making them suitable for implantation of the test miniplates. Bicortical screws can be placed in this region without violating the mandibular canal. The dotted oval indicates the position of the mandibular foramen, located by illuminating the medial aspect of the ramus; dashed lines outline the mandibular canal. (B) Two test miniplates can be fixed to the lateral aspect of each mandible (four per sheep). Image tone and background were modified in Adobe Photoshop 2025.

This technical note reports the development of an ovine model for this purpose. The model permits testing of up to four experimental implants (e.g., biomaterials) per animal using a repeated‐measures design, in which the implant (group) is a fixed effect, animals are a clustering factor, and the surgical site (side) is a fixed or random effect. The model is analogous to those described for the testing of multiple biomaterials for musculoskeletal applications within the same animal.^5^, ^6^, ^7^ The ramus of the ovine mandible is particularly suitable for the testing of biomaterials when a relatively large flat bony surface is required (Figure 1).

Several aspects of ovine maxillofacial anatomy have been described.^8^, ^9^, ^10^, ^11^, ^12^, ^13^, ^14^ However, the surgical approach to the ramus and its relevant anatomy have not yet been detailed for this species. An endoscopic technique for fracture fixation in the subcondylar region of the ovine mandible has been reported, but it lacks anatomical detail and is largely extrapolated from human anatomy.^15^ Surgical approaches described in other species, such as the dog,^16^ are not directly applicable to sheep due to fundamental anatomical differences.

In sheep, the parotid gland is extensive reflecting the functional demands of rumination. Its broad distribution and extremely delicate fascia (capsule) make preservation during dissection challenging,^17^ increasing the risk of unintended intraoperative tears and salivary leakage. Surgical manipulation in this region should therefore consider the risk of sialocele formation.^18^

The aims of this study were to (1) describe the surgical approach to the mandibular ramus in sheep including the relevant regional anatomy, and (2) demonstrate that the proposed model is safe, humane, and feasible.

## 2 MATERIALS AND METHODS

### 2.1 Animals

Five commercially sourced 2‐year‐old castrated male Merino sheep (37–43 kg body weight on arrival) were used for this study. The animals were housed and maintained in the animal house of the Melbourne Veterinary School, The University of Melbourne, and were kept in two pens, with one containing two sheep and the other three. They were provided with a ration of pellets once a day, and dry hay and water were provided ad libitum. An acclimatization period of at least 2 weeks was observed before any procedures were commenced.

### 2.2 Anesthetic protocol

The animals were premedicated using methadone (0.6 mg/kg IM) and midazolam (0.5 mg/kg IM) 15–20 min prior to induction of anesthesia with IV propofol (4–6 mg/kg) titrated to effect. Anesthesia was maintained with 1.5% to 3.5% isoflurane in oxygen. Hair and wool over the caudolateral aspect of both mandibles, with a wide surrounding margin, were clipped and the area was prepared aseptically.

A high inferior alveolar nerve block (extraoral retromandibular needle insertion) was performed using ropivacaine (5 mg adjusted to total 1 mL per side) immediately before surgery to desensitize the bone and regional soft tissues. A 23 gauge, 50 mm long hypodermic needle was inserted at the level of the mandibular angle and directed toward the area dorsal to the mandibular foramen (approximately 10 mm), remaining flush with the medial wall of the mandible (see Figure 1). Defleshed ovine mandibles were used to determine the appropriate insertion angle and depth. Data on the duration of action of ropivacaine on the inferior alveolar nerve in sheep are unavailable. Ropivacaine was selected because of its reportedly longer duration of action compared with lidocaine.^19^

### 2.3 Implant placement

**FIGURE 2:**
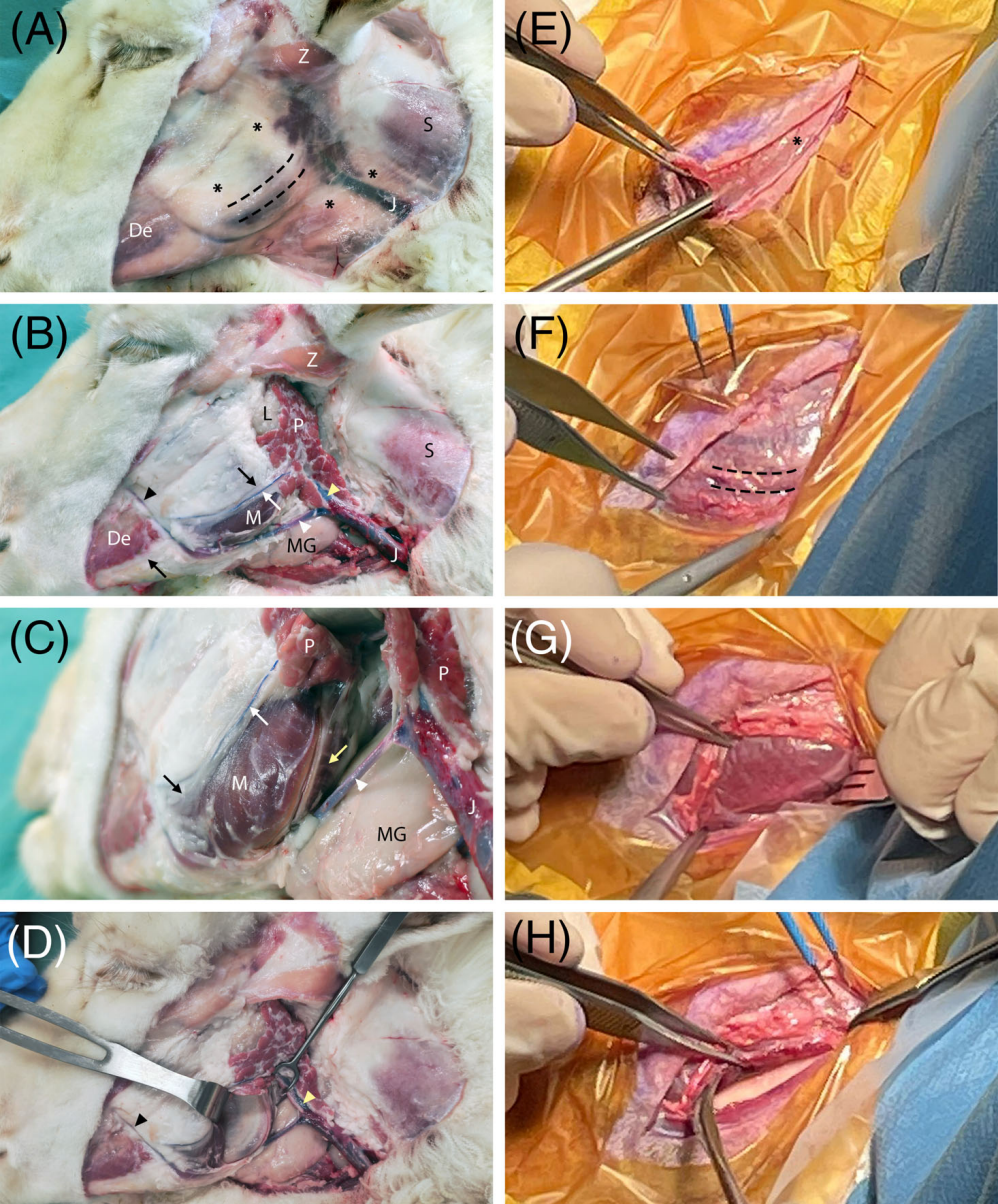
Photo series demonstrating the four key steps of accessing the body and ramus of the left ovine mandible (Australian Merino). (A–D) Staged dissection of the tissues overlying the left mandible in a cadaver specimen (rostral to the left; dorsal to the top). (A) Appearance of the superficial musculoaponeurotic system (platysma and associated fascia; SMAS) following removal of the skin and subcutaneous tissue. (B) Removing the SMAS reveals the parotid gland, mimetic muscles, and deep neurovascular structures. (C) The pterygomasseteric sling, a fibromuscular structure formed by the masseter and medial pterygoid muscles at the mandibular angle, is visualized by separating the parotid glandular tissue carefully from the mandibular gland and underling structures while gently retracting the parotid gland dorsally. A blue suture may be seen within the lumen of the parotid duct (white arrows). The ventral buccal (marginal mandibular) branch of the facial nerve courses within the same neurovascular bundle (between the dashed lines in A) as the parotid duct for the first few centimeters following its emergence from the rostroventral border of the parotid gland (black arrows). More rostrad, this nerve lies on the ventral margin of the depressor labii inferioris. (D) Exposure of the mandible is achieved by subperiosteal elevation of the masseter and its rostral retraction. (E–H) Intraoperative images corresponding to each dissection stage presented on the left. Exposure has been kept to a minimum. (E) A small curved Metzenbaum scissors is used to gently elevate and incise the platysma muscle. (F) This is followed by blunt subparotid dissection to mobilize the gland and its associated neurovascular structures (between the dashed lines) dorsally. (G) This allows direct visualization of the pterygomasseteric sling. (H) Subperiosteal elevation of the masseter muscle reveals the mandible. Black arrowhead, facial vein; white arrowhead, linguofacial vein; yellow arrowhead, maxillary vein; J, external jugular vein; P, parotid gland; MG, mandibular gland; L, parotid lymph node; *, superficial musculoaponeurotic system (SMAS); (M), masseter; yellow arrow, pterygoideus medialis; Z, zygomaticoauricularis; De, depressor labii inferioris; (S), sternocephalicus. Image tone and background have been modified in Adobe Photoshop 2025.

Figure 2 illustrates the retromandibular subparotid approach to the mandibular ramus, and Appendix A provides a detailed description of the method.

The model uses a purpose‐designed additively manufactured test miniplate (approximately 20 mm long, 7.5 mm wide, and 3.5 mm deep), supplied as a .stl file in the Supporting Information. Each miniplate bears an internal cavity (approximately 9 mm long and 5.5 mm wide) that can be filled with the test material. Alternatively, the .stl file could be modified to include an inner geometry allowing for the manufacturing of each miniplate in a different biomaterial.

Two distinct areas on the lateral aspect of each ramus close to the caudal and ventral borders (Figure 1B) were suitable for the placement of test miniplates, permitting up to four miniplates per animal. Appropriate control miniplates should be included in each animal depending on the study design. Alternatively, a single miniplate per side may be used if required.

**FIGURE 3:**
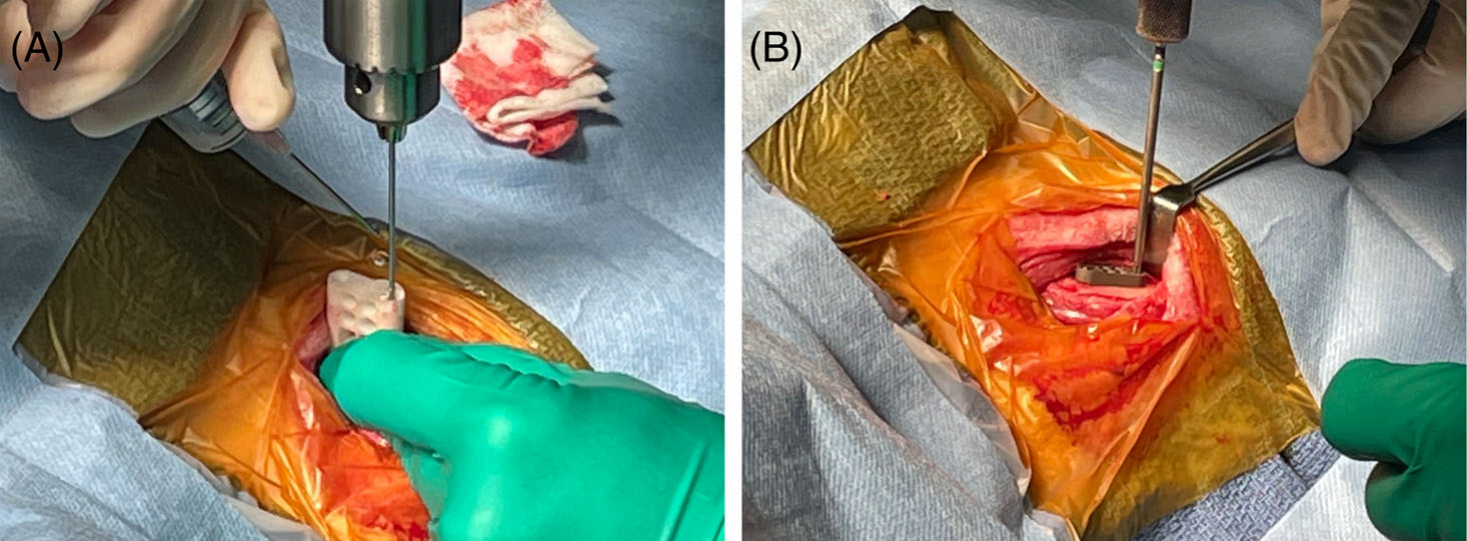
The technique used to apply the miniplates. (A) The custom three‐dimensional (3D) printed (Nylon) guide helps work within a relatively confined space while protecting the soft tissues from the rotating drill bit. (B) The dorsal miniplate is applied first, as dorsally as practicable. A narrow Senn retractor can help create more room for the screwdriver during screw placement.

Due to limited exposure, a custom surgical drill guide (supplied as an .stl file in the Supporting Information) was required for pilot hole preparation (Figure 3A). Pilot holes were drilled with a 1.5 mm drill bit under irrigation. The dorsal miniplate was applied first and placed as dorsally as feasible to allow correct placement of the second miniplate in the more ventral position. Care was taken to avoid unintended pilot holes, as the resulting bony reaction may confound data interpretation. Each miniplate was secured to the mandible with two 2.0 mm self‐tapping titanium screws (OsteoMed Inc., Addison, Tx, USA; Figure 3B). Screw length was determined intraoperatively using a depth gauge to ensure bicortical purchase and secure fixation. Only 10–12 mm screws were required in this study. Overtightening was avoided.

### 2.4 Postoperative period

Postoperative analgesia consisted of meloxicam (1 mg/kg subcutaneously once daily for 6 days, including a preoperative dose) and buprenorphine (0.01 mg/kg subcutaneously twice daily for 4 days, including the day of surgery). Procaine penicillin (25 000 IU/kg IM once daily) was used for 5 days starting on the day of surgery. Monitoring comprised evaluation of the surgical sites and behavioral parameters: facial grimace,^20^ posture (head and neck as well as overall body), vocalization, appetite, feed prehension, mastication, swallowing, and body condition scores using the form provided in Appendix A. Animals were euthanized 24 weeks after surgery by IV pentobarbital overdose.

## 3 RESULTS

All five animals appeared fully weight bearing within 30 min of anesthetic recovery. Eating resumed immediately in all cases with no discernible mastication abnormalities. A maximum grimace score of 1 or 2 (out of 10)^20^ was recorded within the first 48 h of surgery, with scores of 0 afterwards; this prompted immediate analgesic supplementation. No infections, wound‐healing complications, or evidence of breakthrough pain were observed during the study. Of the 10 surgical sites, two sites in two animals (one per animal) developed mild swellings within 48 h of the procedure. The swellings were superficially soft and undulating, with a small, firm core palpable at depth, suggestive of small sialoceles or seromas. They were not painful on palpation and were not associated with drainage. Body temperature remained normal throughout. Given their small size and lack of clinical significance, the swellings were not further investigated and resolved spontaneously within 8 weeks postoperatively.

Body condition scores remained constant (3 out of 5) in all animals. Postmortem examination at 24 weeks postoperatively revealed no gross abnormalities, and all surgical sites were fully healed with minimal to no visible scar formation.

## 4 DISCUSSION

The model provides a safe experimental tool for evaluating biomedical implants in the maxillofacial region. Up to four different test and control groups can be assessed per animal, reducing the total number of animals required in accordance with the principle of “reduction” in experimental design. This arrangement also permits the use of repeated measures statistics, further decreasing animal numbers by increasing statistical power. Caution is advised when designing experiments in which the regional acceleratory phenomenon^21^ could confound results due to the close proximity of implants.

Postmortem histological or microbiological evaluations are not reported here, as these should be performed and interpreted in the context of the specific biomaterial or technology tested. The anesthesia and analgesia protocol, the surgical approach, and preoperative and postoperative care preserve important anatomical structures, support excellent recovery, and minimize overall experimental burden on the animals in accordance with the principle of “refinement.” A small risk of surgical site swelling (likely sialocele or seroma) was observed; this was minimally disruptive and self‐limiting, with the masseter muscle providing a thick barrier that protects implants and surrounding tissues. Nevertheless, researchers are advised to assess any postoperative swelling, including through the use of ultrasound, to ensure implant integrity.

## AUTHOR CONTRIBUTIONS

Reza Sanaei: Conceptualization, investigation, methodology, visualization, writing—original draft. Ayda Farhoudi and Babatunde A. Ayodele: Data curation, investigation, writing—review and editing. Helen M. Davies: Conceptualization, investigation, methodology, writing—review and editing. George Dimitroulis, Christina M. Murray, Alastair J. Sloan, and Charles N. Pagel: Conceptualization, methodology, writing—review and editing.

## FUNDING INFORMATION

This research did not receive any specific grant from funding agencies in the public, commercial, or not‐for‐profit sectors. MAXONIQ Australia provided in‐kind contributions of time and resources during the experimental design stage of the work.

## CONFLICT OF INTEREST

George Dimitroulis is the founder of MAXONIQ Australia. This model was developed for the purpose of preclinical testing of maxillofacial implants designed by MAXONIQ. Reza Sanaei and Alastair J. Sloan had a formal agreement with MAXONIQ to evaluate the implants. The remaining authors declare no conflict of interest.

## Supporting information


**Data S1.** Supporting Information.

## APPENDIX A

### A.1 DETAILED DESCRIPTION OF THE RETROMANDIBULAR SUBPAROTID APPROACH TO THE RAMUS OF OVINE MANDIBLE

The animals were placed in lateral recumbency with the proposed incision site uppermost. The skin incision began approximately 2 cm ventral to the base of the ear and extended ventrally for 4–5 cm in a curvilinear line roughly following the caudal border of the mandible (Figure 2), maintaining a 1.5 cm distance from the mandibular margin along its length. In standing animals, after recovery from surgery, this incision line appeared displaced ventrally and rostrally, reflecting the abundant loose skin in this region in the Merino.

The superficial fascia was sharply incised on the same line as the skin. A small amount of subcutaneous/preplatysmal fat was encountered and bluntly dissected to expose the superficial musculoaponeurotic system (SMAS), mainly the platysma in this region. The platysma was lifted and sharply incised along the skin line. To protect the underlying parotid gland and neurovascular structures, the platysma was first tented and nicked, followed by subplatysmal blunt dissection to separate the muscle from underlying tissues before completing the incision (Figure 2E).

The parotid gland in sheep is quite extensive, somewhat delicate, and contained within a very fine connective tissue capsule (Figure 2B); care was taken to avoid incising the gland. Any breaches were promptly closed using USP 3–0 or 4–0 polyglactin 910 (Vicryl, ETHICON, Australia) in a mattress or cruciate pattern. The gland is closely associated with the external jugular vein and its tributaries, including the linguofacial and maxillary veins (Figure 2B). The parotid duct and the ventral buccal branch of the facial nerve were identified and protected at this stage.

Blunt dissection into the subplatysmal fat and areolar connective tissue was performed ventral to these structures (Figure 2F) with the aim of identifying the ventral border of the parotid gland. Once identified, a subparotid dissection plane was developed between the parotid gland and the linguofacial vein (Figure 2F). A number of small vessels and the cervical branch of the facial nerve may be encountered here. These do not need to be spared for a successful postoperative recovery; however, the decision to preserve the cervical branch of the facial nerve should be made based on the purpose of the surgery, anatomic variation, and space required. No attempt was made to preserve the cervical branch of the facial nerve in this study. This did not seem to lead to any postoperative complications as the skin in this region of Merinos remains folded and somewhat pendulous.

Once the ventral parts of the parotid gland, containing the maxillary vein, were sufficiently mobilized, dissection proceeded to identify the pterygomasseteric sling and the caudoventral border of the mandible (Figure 2G). The pterygomasseteric sling is the fibromuscular envelope formed by the insertions of the masseter and medial pterygoid muscles on the mandibular angle. Dorsal retraction of the parotid gland is necessary during this stage.

Generous exposure of the masseter surface facilitates later closure of the pterygomasseteric sling as the masseter tends to retract considerably after subperiosteal dissection to expose the masseteric fossa. The ventral masseteric branch of the maxillary vein was often visible on the lateral masseter surface and could usually be preserved. In two cases, a relatively large vein entering the masseter caudoventrally had to be isolated, ligated, and transected before elevating the muscle. This vein may be mistaken for the facial vein based on human anatomy; however, in sheep, the facial vein is a tributary of the linguofacial vein, running dorsoventrally rostral to the masseter before following the ventral mandibular border to join its lingual counterpart and form the linguofacial vein. The linguofacial vein then courses ventrally and medially relative to the mandible before joining the maxillary vein near the caudoventral border of the parotid gland to form the external jugular vein.

A no. 15 scalpel blade was used to incise the pterygomasseteric sling and the periosteum at the caudoventral border of the mandible. Subperiosteal dissection of the masseter muscle was then performed using a periosteal elevator to expose the mandibular ramus (Figure 2H). Diffuse bleeding at this stage was controlled by pressure. Heavy retraction, for example with an army‐navy retractor, may be required to visualize and access the ramus, particularly during drilling. Use of a custom three‐dimensional (3D) printed surgical guide (.stl file supplied) and a right‐angle surgical drill is highly beneficial to minimize trauma to the overlying parotid gland and other soft tissues during implant placement.

Following completion of the procedure, the pterygomasseteric sling was closed with three mattress sutures using a monofilament synthetic absorbable suture (USP 3–0 polydioxanone, PDS II, ETHICON). Care was taken to avoid including parotid tissue. If required, subplatysmal fat may be approximated to obliterate dead space, with extreme caution to avoid parotid tissue inclusion; in this study, this layer was not closed.

The platysma was closed in a running pattern using USP 3–0 PDS II to provide a watertight seal against any potential salivary leakage. A surgical drain may be placed before skin closure, although the risk of an ascending infection is elevated depending on husbandry conditions. In our experience, successful outcomes were achieved without drains despite the loose skin typical of Merinos.

Subcutaneous tissue and skin were closed in two separate layers. A simple interrupted pattern with buried knots was used for the subcutaneous tissue and a continuous intradermal pattern for the skin using a 4/0 polyglecaprone 25 (Monocryl, ETHICON), avoiding the need for later suture removal. Following skin closure on the first side, the area was gently cleaned with moist lint‐free gauze and normal saline and the animal repositioned in preparation for the surgery on the opposite side. The first incision was protected with loose sterile dressing during the second procedure. No wound dressings were applied postoperatively.

In this study, implantation of two miniplates per side required 30–40 min (skin to skin) with shorter durations achieved in later procedures as technique and operator confidence improved.

## APPENDIX B

### B.1 MONITORING SHEETS

**Table:**

Signs	Day 0		Day 1			Day 2			Day 3		
Local reactions *(If sign present, mark field “✓,” if not mark “✘”)*	1	2	1	2	3	1	2	3	1	2	3
Visible swelling											
Redness (hyperemia and congestion)											
Hemorrhage											
Discharge											
Dehiscence/Wound breakdown											
Signs of distress/pain (*If sign present, mark field “✓*,” *if not mark “✘”*)											
Change in feeding											
Abnormal head carriage											
Abnormal mastication or loss thereof											
Behavioral changes, e.g., depression, lethargy											
Vocalization											
Facial expression score (out of 10)^12^											
Analgesics (*mark with a* “*✓*” *after administration*)											
Meloxicam (1 mg/kg SC sid)—dose given:											
Buprenorphine (0.01 mg/kg SC)—dose given:											
**Time monitoring performed (hh:mm)**											
**Investigator (initials)**											
**Comments** *Provide detail of any signs, body weight or condition changes, or other observations of note*.											

**STAGE 1: INTENSIVE IMMEDIATE POSTOPERATIVE CARE AND MONITORING (DAY 0 TO DAY 3 POSTOP)**

**GENERAL INFORMATION**

**Date (implantation performed):**

**Ethics ID:**

**Species:** Sheep

**Breed:** Merino

**Animal ID:**

**Procedure:** Bilateral mandibular miniplate implantation

**OTHER DRUGS**

**STAGE 2: EARLY POSTOPERATIVE CARE AND MONITORING (DAY 4 TO 14 POSTOP)**

**Table:**

Name	Dose (mg)	Route	Date	Time	Reason
1.					
2.					
3.					

**GENERAL INFORMATION**

**Date (day 4):**

**Ethics ID:**

**Species:** Sheep

**Breed:** Merino

**Animal ID:**

**Procedure:** Bilateral mandibular miniplate implantation

**DRUGS**

**Table:**

Name	Dose (mg)	Route	Date	Time	Reason
1.					
2.					
3.					

Day Local reactions (*If sign present, mark field* “*✓*,” *if not mark* “*✘*”)	4		5		6		7		8		9		10		11		12		13		14	
Visible swelling																						
Redness (hyperemia and congestion)																						
Hemorrhage																						
Discharge/fistulation																						
Dehiscence/Wound breakdown																						
Signs of distress/pain (*If sign present, mark field* “*✓*,” *if not mark* “*✘*”)																						
Change in feeding																						
Abnormal head carriage																						
Abnormal mastication or loss thereof																						
Behavioral changes, e.g., depression, lethargy																						
Pacing																						
Vocalization																						
Facial expression score (out of 10)																						
General wellbeing																						
Body condition score (out of 5)																						
**Time monitoring performed (hh:mm)**																						
**Investigator (initials)**																						
**Comments** *Provide detail of any signs, body weight or condition changes, or other observations of note*.																						

**Stage 3: LONG‐TERM POSTOPERATIVE MONITORING (DAYS 15 TO 40)**

**GENERAL INFORMATION**

**Date (day 15):**

**Ethics ID:**

**Species:** Sheep

**Breed:** Merino

**Animal ID:**

**Procedure:** Bilateral mandibular miniplate implantation

**DRUGS**

**Table:**

Name	Dose (mg)	Route	Date	Time	Reason
1.					
2.					
3.

**Table:**

Day Local reactions *(If sign present, mark field “✓,” if not mark “✘”)*	15	16	17	18	19	20	21	22	23	24	25	26	27	28	29	30	32	34	36	38	40
Visible swelling																					
Redness (hyperemia and congestion)																					
Hemorrhage																					
Discharge/fistulation																					
Dehiscence																					
Signs of distress/pain (*If sign present, mark field “✓*,” *if not mark* “*✘*”)																					
Change in feeding																					
Abnormal head carriage																					
Abnormal mastication or loss thereof																					
Behavioral changes, e.g., depression, lethargy																					
Pacing																					
Vocalization																					
Facial expression score (out of 10)																					
General wellbeing																					
Body condition score (out of 5)																					
**Time monitoring performed (hh:mm)**																					
**Investigator (initials)**																					
**Comments** *Provide detail of any signs, body weight or condition changes, or other observations of note*.																					

**Stage 3: LONG‐TERM POSTOPERATIVE MONITORING (DAY 42 TO WEEK 14)**

**GENERAL INFORMATION**

**Date (day 36):**

**Ethics ID:**

**Species:** Sheep

**Breed:** Merino

**Animal ID:**

**Procedure:** Bilateral mandibular miniplate implantation

**Table:**

Name	Dose (mg)	Route	Date	Time	Reason
1.					
2.					
3.					

**DRUGS**

**Table:**

Day/week Local reactions (*If sign present, mark field* “*✓*,” *if not mark “✘*”)	42	44	46	48	50	52	54	56	58	60	62	wk 10		wk 11		wk 12		wk 13		wk 14	
												1	2	1	2	1	2	1	2	1	2
Visible swelling																					
Redness (hyperemia and congestion)																					
Hemorrhage																					
Discharge/fistulation																					
Dehiscence																					
Signs of distress/pain (*If sign present, mark field* “*✓*,” *if not mark* “*✘*”)																					
Change in feeding																					
Abnormal head carriage																					
Abnormal mastication or loss thereof																					
Behavioral changes, e.g., depression, lethargy																					
Pacing																					
Vocalization																					
Facial expression score (out of 10)																					
General wellbeing																					
Body condition score (out of 5)																					
**Time monitoring performed (hh:mm)**																					
**Investigator (initials)**																					
**Comments** *Provide detail of any signs, body weight or condition changes, or other observations of note*.																					

**Stage 3: LONG‐TERM POSTOPERATIVE MONITORING (WEEK 15 TO WEEK 25)**.

**GENERAL INFORMATION**

**Date (day 36):**

**Ethics ID:**

**Species:** Sheep

**Breed:** Merino

**Animal ID:**

**Procedure:** Bilateral mandibular miniplate implantation

**DRUGS**

**Table:**

Name	Dose (mg)	Route	Date	Time	Reason
1.					
2.					
3.

**Table:**

DaysLocal reactions (*If sign present, mark field* “*✓*” *if not mark* “*✘*”)	wk 15		wk 16		wk 17		wk 18		wk 19		wk 20		wk 21		wk 22		wk 23		wk 24		wk 25	
	1	2	1	2	1	2	1	2	1	2	1	2	1	2	1	2	1	2	1	2	1	2
Visible swelling																						
Redness (hyperemia and congestion)																						
Hemorrhage																						
Discharge/fistulation																						
Dehiscence																						
Signs of distress/pain (*If sign present, mark field* “*✓*,” *if not mark* “*✘*”)																						
Change in feeding																						
Abnormal head carriage																						
Abnormal mastication or loss thereof																						
Behavioral changes, e.g., depression, lethargy																						
Pacing																						
Vocalization																						
Facial expression score (out of 10)																						
General wellbeing																						
Body condition score (out of 5)																						
**Time monitoring performed (hh:mm)**																						
**Investigator (initials)**																						
**Comments** ***Provide detail of any signs, body weight or condition changes, or other observations of note***																						

**Date euthanized:**

INTERVENTION CRITERIA

**Severity Table**

Use the table below to determine the severity of the observations recorded in the associated monitoring sheet. If necessary, seek independent veterinary advice.

**Table:**

Signs	Mild to moderate	Severe
Local reactions		
Visible swelling	Slight swelling expected	Sutures stretched or large swelling
Redness (hyperemia and congestion)	Slight redness is expected	Wound margins seem abnormally red
Hemorrhage	Some intermittent small drops of blood or oozing may be expected (<10 mL in total)	Uncontrolled bleeding or a continuous stream
Discharge/fistulation	Small amount of clear discharge/crusting noticeable	Excessive discharge, colored discharge or obvious pustulation/abscessation
Wound healing/dehiscence	Wound healing slower than expected, i.e., it will likely take more than 14 days to completely heal.	Wound healing not progressed, dehiscence evident
Any sign of distress/pain		
Change in feeding	Slight loss of appetite, 15% feed refusal	More than 15% refusal up to complete loss of appetite
Abnormal head carriage (dorsal, ventral, rotational, etc.)	Abnormal carriage barely noticeable	Abnormal carriage clearly noticeable
Abnormal mastication or loss thereof	Infrequent slight disruptions of unilateral or bilateral mastication	Frequent/obvious disruption of mastication
Pacing	Slightly increased	Obviously increased or constantly pacing
Behavior	Animal slightly agitated or depressed	Agitated or depressed
Facial expression score^12^	A score of 5 or below	A score of 6 or more
General wellbeing		
Body condition score	Body condition score between 2 and 3	Body condition score of 2 or less

**Intervention Criteria Table**

**Table:**

Criteria	Action required
3 or less mild to moderate local reaction signs (days 0 to 14)	Increase monitoring frequency to 4 times daily. Gently clean the area if appropriate. If additional analgesics are considered or used, contact an independent veterinarian.
4 or more mild to moderate local reaction signs (day 0 to 14)	Seek independent veterinary advice.
1 or more mild to moderate local reaction sign (day 15 onwards)	Seek independent veterinary advice.
2 or less mild to moderate signs of distress/pain	Increase monitoring frequency by an additional check. Give analgesics and seek independent veterinary advice.
3 or less mild to moderate signs of distress/pain	Seek independent veterinary advice.
Mild to moderate loss of body condition score	Increase feed and increase monitoring frequency to once every 72 h.
1 severe sign	Seek independent veterinary advice.
